# Nuclear pore complexes undergo Nup221 exchange during blood-stage asexual replication of *Plasmodium* parasites

**DOI:** 10.1128/msphere.00750-24

**Published:** 2024-11-11

**Authors:** James Blauwkamp, Sushma V. Ambekar, Tahir Hussain, Gunnar R. Mair, Josh R. Beck, Sabrina Absalon

**Affiliations:** 1 Department of Pharmacology and Toxicology, Indiana University School of Medicine, Indianapolis, Indiana, USA; 2 Department of Biomedical Sciences, Iowa State University, Ames, Iowa, USA; 3 School of Biological Sciences, Queen’s University Belfast, Belfast, United Kingdom; Virginia-Maryland College of Veterinary Medicine, Blacksburg, Virginia, USA

**Keywords:** *Plasmodium berghei*, nuclear pore complexes, expansion microscopy, RITE system

## Abstract

*Plasmodium* parasites, the causative agents of malaria, undergo closed mitosis without breakdown of the nuclear envelope. Unlike closed mitosis in yeast, *Plasmodium berghei* parasites undergo multiple rounds of asynchronous nuclear divisions in a shared cytoplasm. This results in a multinucleated organism prior to the formation of daughter cells within an infected red blood cell. During this replication process, intact nuclear pore complexes (NPCs) and their component nucleoporins play critical roles in parasite growth, facilitating selective bi-directional nucleocytoplasmic transport and genome organization. Here, we utilize ultrastructure expansion microscopy to investigate *P. berghei* nucleoporins at the single nucleus level throughout the 24-hour blood-stage replication cycle. Our findings reveal that these nucleoporins are distributed around the nuclei and organized in a rosette structure previously undescribed around the centriolar plaque, responsible for intranuclear microtubule nucleation during mitosis. By adapting the recombination-induced tag exchange system to *P. berghei* through a single plasmid tagging system, which includes the tagging plasmid as well as the Cre recombinase, we provide evidence of NPC formation dynamics, demonstrating Nup221 turnover during parasite asexual replication. Our data shed light on the distribution of NPCs and their homeostasis during the blood-stage replication of *P. berghei* parasites.

**IMPORTANCE:**

Malaria, caused by *Plasmodium* species, remains a critical global health challenge, with an estimated 249 million cases and over 600,000 deaths in 2022, primarily affecting children under five. Understanding the nuclear dynamics of *Plasmodium* parasites, particularly during their unique mitotic processes, is crucial for developing novel therapeutic strategies. Our study leverages advanced microscopy techniques, such as ultrastructure expansion microscopy, to reveal the organization and turnover of nuclear pore complexes (NPCs) during the parasite’s asexual replication. By elucidating these previously unknown aspects of NPC distribution and homeostasis, we provide valuable insights into the molecular mechanisms governing parasite mitosis. These findings deepen our understanding of parasite biology and may inform future research aimed at identifying new targets for anti-malarial drug development.

## INTRODUCTION

**Fig 1:**
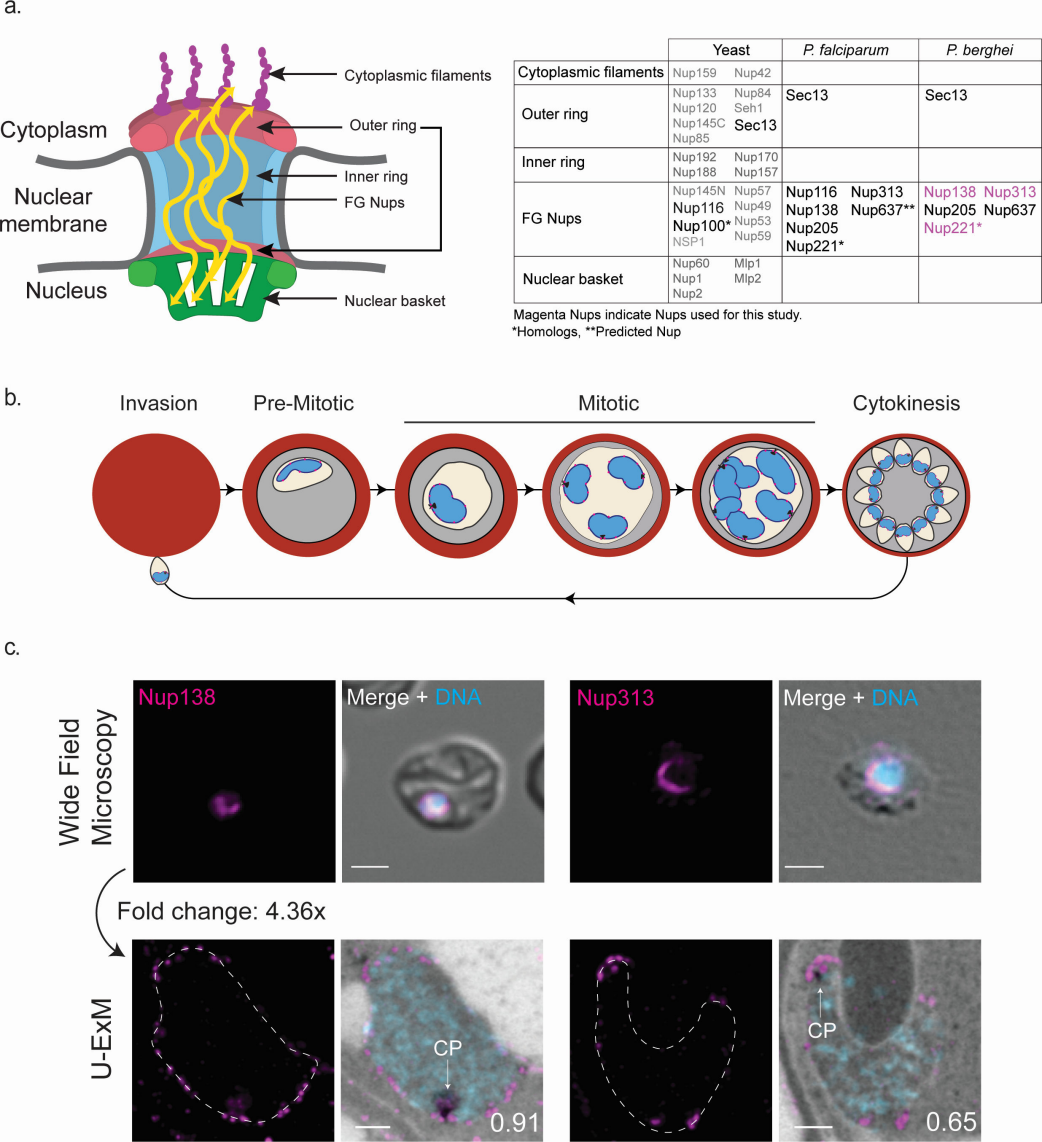
Distribution of divergent *Plasmodium berghei* NPCs can be captured by ultrastructure expansion microscopy (U-ExM). (a) Schematic representation and table of known nucleoporins in yeast, *Plasmodium falciparum*, and *P. berghei*. Nups highlighted in magenta indicate Nups visualized in this study, Nups marked with an asterisk represent homologs, Nup637 predicted Nup. (b) Schematic representation of the *P. berghei* life cycle inside host red blood cells. Parasites begin with a single nucleus that undergoes mitosis midway through the life cycle. Multiple rounds of asynchronous mitosis led to a multinucleated parasite with a single cytoplasm before a shared cytokinetic event produced individual parasites. Blue is DNA, magenta dots represent NPCs. (c) Comparisons between unexpanded and U-ExM images of Nup138::smHA and Nup313::smHA (magenta), with DNA shown in blue and DIC for widefield or protein density for U-ExM shown in grayscale. Scale bars 2 µm; the number on images indicates image depth in micrometer.

The survival and growth of eukaryotic organisms rely on the movement of macromolecules between the membrane-bound nucleus and the cytosol. This nuclear-cytosolic transport is facilitated by nuclear pore complexes (NPCs) that are embedded into the nuclear envelope. Apart from acting as a selective barrier for bidirectional nucleocytoplasmic transport, the NPC plays a key role in chromatin organization, gene regulation, DNA repair, and maintenance of epigenetic memory in many eukaryotes (1). Typically, these NPCs are composed of multiple copies of more than 30 different nucleoporins (Nups) per NPC and contain up to 1,000 individual proteins organized in an eightfold symmetry (1–5). Nups are widely conserved in most eukaryotes, including yeasts, humans, and various plant species and have also been identified in parasitic protozoa such as *Trypanosoma brucei* and *Toxoplasma gondii* (6, 7). Nups are separated into two distinct categories: structural Nups that form the pore and the intrinsically disordered phenylalanine-glycine repeat (FG) Nups, which regulate nucleocytoplasmic transport (Fig. 1a) (8, 9). Structurally, the NPCs establish an inner channel called the inner ring, flanked by the outer rings on the cytosolic and nuclear sides of the NPC. After the inner channel is constructed, the cytoplasmic filament and the nuclear basket Nups attach to their appropriate sides of the pore (8, 9). The FG Nups, characterized by their FG repeats, occupy the space inside the inner ring and interact with the cargo to facilitate nuclear-cytosolic transport (8–10).

Budding yeast is one of the most extensively studied organisms with respect to the dynamics and structural aspects of nuclear pore complexes. *Saccharomyces cerevisiae* undergoes closed mitosis during which the nuclear envelope remains intact during karyokinesis. In contrast to open mitosis, where NPC removal during prophase leads to nuclear envelope breakdown, in closed mitosis, it has been hypothesized that specific regions of nuclear pore complex disassembly cause small breaks in the nuclear envelope, facilitating nuclear envelope fission and formation of two new nuclei (11, 12). Like budding yeast, the parasites of the *Plasmodium* genus, the causative agent of malaria, undergo a specialized form of closed mitosis. In these parasites, multiple rounds of asynchronous nuclear division in a shared cytoplasm lead to a multinucleated single-celled parasite (13). While many Nups are conserved across evolution, *Plasmodium* Nups are highly divergent. Their identification by bioinformatics has, therefore, proven challenging. In a comprehensive phylogenetic study of 60 eukaryotes, only two Nups were identified in *P. berghei* compared to 19 identified Nups in over 50 different organisms (14). Eleven Nups are currently known in the rodent malaria model *P. berghei*: Sec13, five FG Nups, and five novel Nups identified using Nup313 as bait in proximal labeling assays (Fig. 1a) (15–17). Interestingly, only one of the novel Nups identified by Ambekar et al. (17) presents established structural features of eukaryotic Nups, further exemplifying the challenges of Nup identification and characterization in *Plasmodium*.

The limited understanding of essential components of nuclear pore complexes in *Plasmodium* is attributed to the absence of protein homology for Nup identification in *Plasmodium* and the lack of suitable tools to investigate the dynamics of NPCs within their comparatively small nuclei (1 µm diameter). While conventional fluorescence and electron microscopy techniques have provided valuable insights into the perinuclear localization of identified Nups in *Plasmodium* parasites, fluorescence microscopy suffers from low resolution, while electron microscopy, despite its higher resolution, requires significant financial and time investments (8, 11, 15, 18–22). These challenges in comprehending the dynamics and distribution of nuclear pore complexes in *Plasmodium* parasites have spurred the exploration of innovative microscopy techniques to overcome these limitations and gain deeper insights into these vital components. The adaptation of ultrastructure expansion microscopy (U-ExM) to work with parasites now allows for super-resolution imaging of parasites in a cost-effective and high-throughput manner (23–26). U-ExM achieves isotropic expansion of biological samples, increasing their size up to 4.5 times by embedding denatured samples into a hydrogel that expands when exposed to water. Notably, this technique has been employed to achieve sub-nucleus resolution and has successfully visualized Nup313 at the nuclear membrane in *Plasmodium falciparum* (23, 25).

In this study, we employed U-ExM to visualize individual NPCs using tagged versions of *P. berghei* Nup138, Nup221, and Nup313. We successfully localized these Nups to the nuclear envelope by employing NHS ester staining throughout the asexual life cycle of the *P. berghei* parasite. Consistent with prior findings using “serial surface” imaging in focused ion beam-scanning electron microscopy (FIB-SEM) (21), we observed an increase in nuclear pores prior to the parasite’s first mitosis, during which the NPCs were then distributed between the newly developing nuclei. We assessed NPC assembly and protein turnover using the Cre-lox recombination induced tag exchange (RITE) system marking the first time this has been accomplished in *Plasmodium*. The RITE system uses a conditional, hormone-activated Cre-recombinase to generate a permanent epitope tag switch in a target coding sequence. This method has been previously utilized in human cells to observe the dynamic replacement of NPC components in human neurons (27, 28).

Leveraging the enhanced resolution of U-ExM coupled with the Cre-lox RITE system, we could monitor NPC assembly and maintenance dynamics within a single nucleus during *Plasmodium berghei* replication. In line with previous observations in mammalian cells (29), we found that NPCs in *P. berghei* undergo protein maintenance after the NPCs have been produced. Remarkably, we discovered the presence of Nup221 proteins of different ages coexisting within a single NPC, as demonstrated by the colocalization of original and newly induced Nup221 signals.

These findings build on existing knowledge and highlight the novel insights that can be gained by integrating U-ExM with genetic and biochemical approaches.

## RESULTS

### U-ExM visualization of Nups around the nucleus

Previous imaging studies on NPCs in various organisms have predominantly relied on slide-based immunofluorescence assays. For higher resolution, NPCs could be visualized by electron microscopy, a time-consuming and costly process. The emergence of expansion microscopy in smaller organisms like yeast has significantly advanced NPC visualization and study (30). In our efforts to visualize nucleoporins at single NPC resolution in *P. berghei*, we introduced C-terminal spaghetti monster Hemagglutinin-3xHA (smHA-3xHA) tags to Nup138 and Nup313, and a 3xHA-GFP tag to Nup221 (Fig. S2). In these transgenic lines, the endogenously tagged genes are the sole source for these Nups in the haploid parasite.

**Fig 2:**
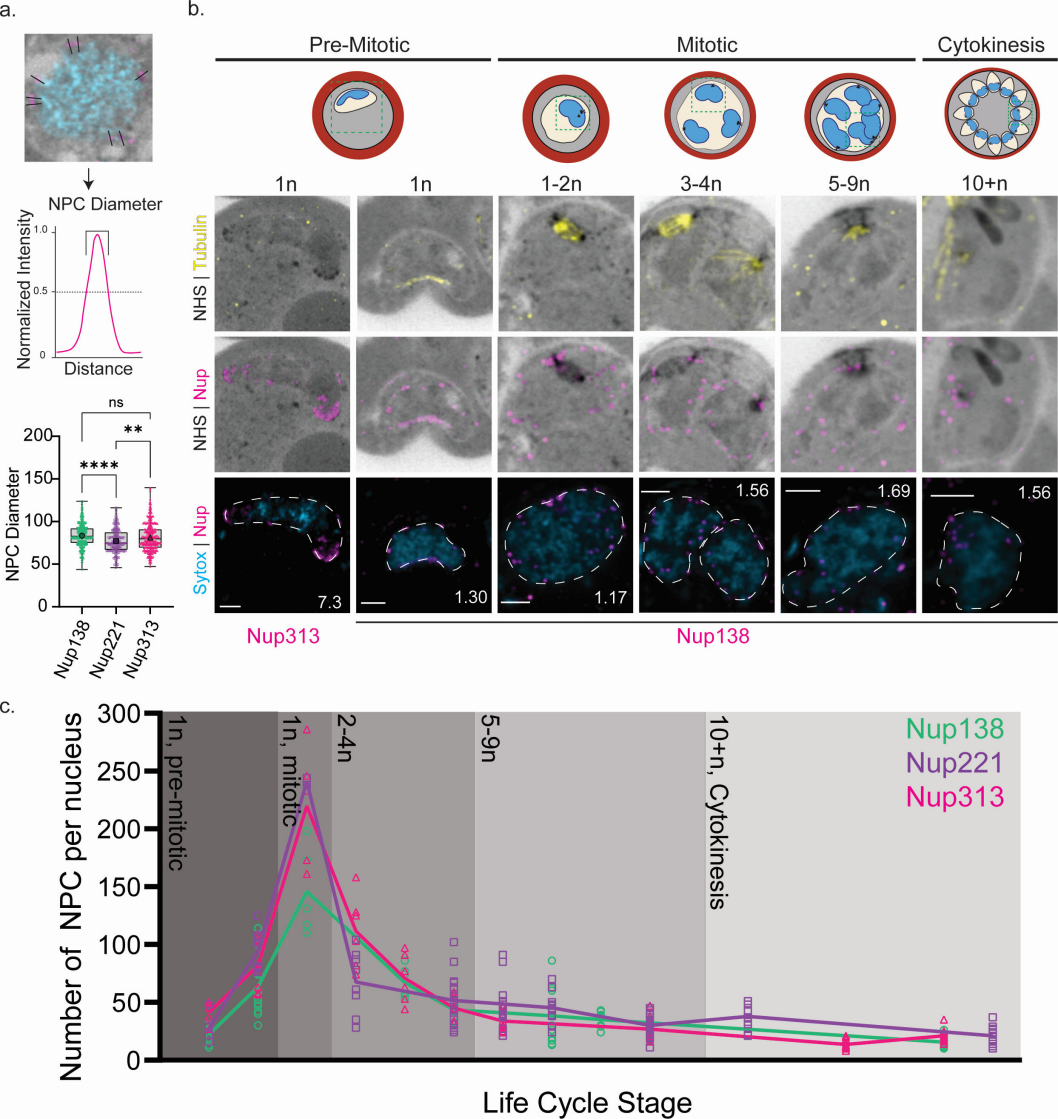
*P. berghei* produces NPCs until mitosis, distributing them among replicated nuclei. (a) Schematic representation of how NPC diameter was determined. A line was drawn through the Nup foci and the intensity normalized to itself. The distance of half the maximal intensity was determined. Shown are the diameters of NPCs measured in cells stained with Nup138::smHA, Nup221::3xHA, and Nup313::smHA. Average size of NPC is indicated by the larger symbol in each column. ****P-value < 0.0001 and **P-value < 0.005. Nup138 *n* = 558 NPC, Nup221 *n* = 188 NPC, Nup313 *n* = 449 NPC. (b) U-ExM images show NPC distribution through the parasite life cycle. Top images: Nup313-smHA at early stages is shown in pink. Images below these show Nup138-smHA through the life cycle of *P. berghei*. Microtubules are shown in yellow, Nup138-smHA is shown in magenta, DNA is shown in blue, and protein density is shown in grayscale. Scale bars 2 µm; the number on images indicate image depth in micrometer. (c) Number of NPCs per nucleus through the *P. berghei* life cycle calculated by counting Nup138::smHA, Nup221::3xHA, and Nup313::smHA signal. Green indicates Nup138 signal, purple indicates Nup221 signal, and magenta indicates Nup313 signal.

In comparison to previous studies using conventionally processed, unexpanded parasites (15, 17), and considering the expected size of the NPCs in yeast (30), expansion microscopy successfully resolves individual NPCs around the nuclear periphery (Fig. 1b and 2a). Based on the size of the gels and the size of the original coverslip, we were able to get an average expansion of 4.36-fold (Fig. S1a). To confirm the cellular position of these Nups at the nuclear membrane, we visualized the nuclear membrane using an endoplasmic reticulum (ER) antibody (PfBIP), employed membrane stains such as Bodipy TR Ceramide and Bodipy FL Ceramide, and assessed membrane localization through NHS ester staining variations around the nucleus (Fig. S1b). We chose to use differences in NHS ester staining to delineate the nuclear envelope and confidently assign the NPC signal to this structure. This allows us to visualize the nuclear envelope without using additional stains. In summary, U-ExM allows us to observe individual NPCs on the nuclear envelope.

### *Plasmodium berghei* produces NPCs primarily before mitosis

As mentioned previously, *Plasmodium* parasites undergo multiple rounds of closed mitosis during their intraerythrocytic development cycle, resulting in a multinucleated organism. As NPC dynamics during closed mitosis are poorly understood, we began by quantifying the number of NPCs surrounding each nucleus during various stages of the parasite’s intraerythrocytic development cycle. To achieve this, we performed U-ExM on parasites that exhibited a range of nuclear numbers and were stained for Nup138::smHA, Nup313::smHA, or Nup221::3xHA. The NPCs in the images collected were then counted by hand for each Z-slice acquired around each nucleus, and the data were plotted against the total number of nuclei in the parasites and the parasite stage. In the early stages of the parasite’s life cycle, before the parasites initiate their mitotic phase (1n, Ring), Nup138 was distributed around the nucleus (Fig. 2b). In contrast, the Nup313 signal was concentrated at opposite ends of the nucleus (Fig. S3). At the single nucleus stage, we noticed a higher number of Nup313 foci (approximately 40–50 NPCs) compared to Nup138 (around 25–35 NPCs) (Fig. 2c). As the parasites began mitosis (1n, Trophozoite pre-mitotic and mitotic), there was a significant fivefold increase in both Nup138 and Nup313 numbers. The parasites produce over 250 NPCs containing Nup313 and over 150 NPCs containing Nup138, distributed around the single nucleus (Fig. 2c). As more nuclei are formed (2–4n Schizont, 5–9n Schizont, 10+n Cytokinesis), the number of NPCs around each nucleus decreased. Initially, approximately 250 NPCs surrounded a single nucleus, which then reduced to around 90–100 NPCs around each of the two or three nuclei. Eventually, the count further decreased to approximately 25–30 NPCs around 10–12 nuclei (Fig. 2c). Our data suggest that NPC production peaks before mitosis, and NPCs are then distributed during each karyokinesis.

### *Plasmodium berghei* NPCs are organized around the centriolar plaque

**Fig 3:**
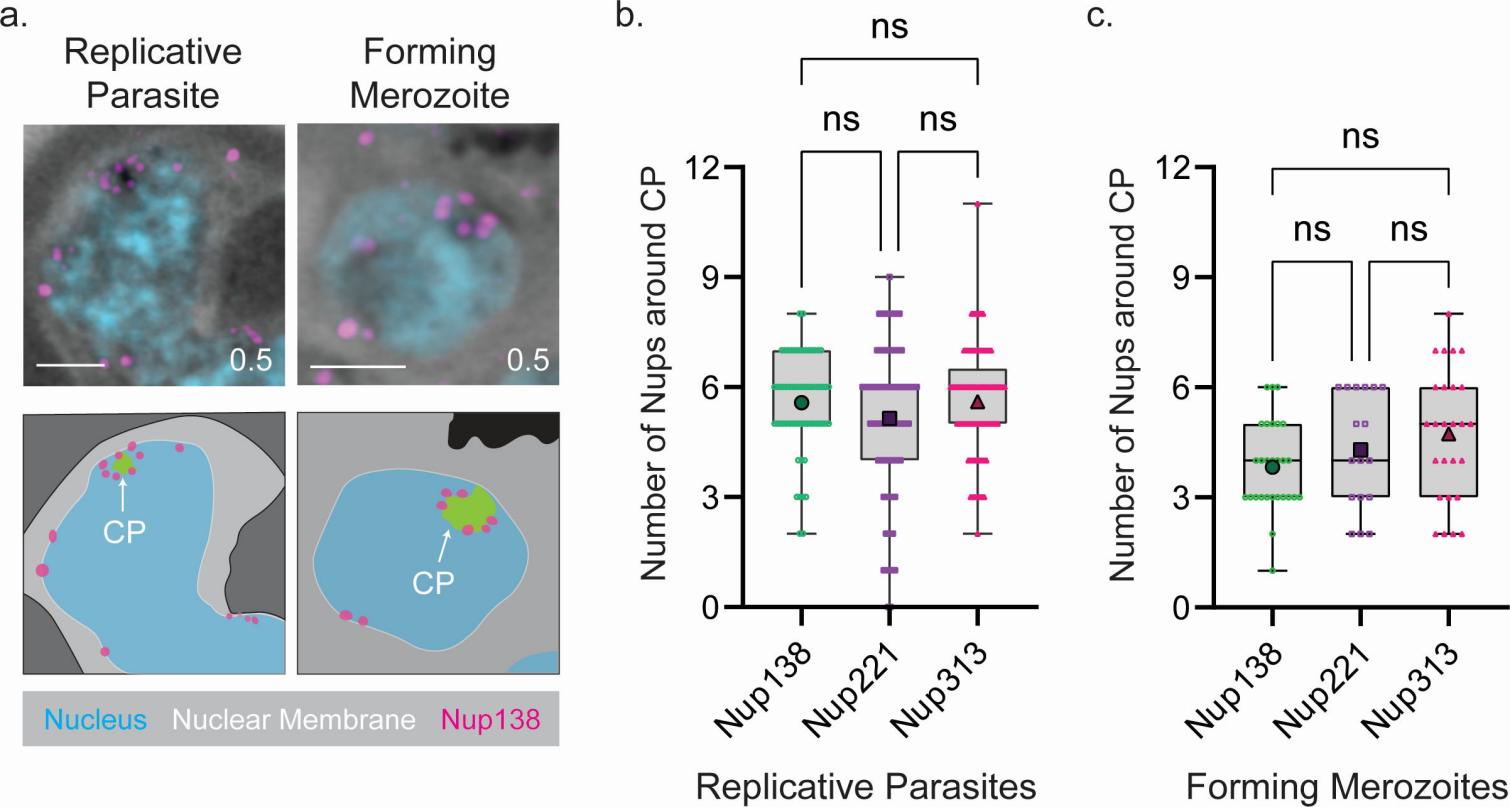
*P. berghei* NPC nuclear arrangements during parasite growth and cytokinesis. (a) Representative images and schematic diagram of Nup138 (magenta) during parasite growth (replicative) and during cytokinesis (forming merozoite) showing Nup138 localization around the CP. Magenta signal that we have reason to believe is background is excluded from the schematic. DNA is shown in blue, protein density is shown in grayscale. Scale bar 2 µm; the number on images indicates image depth in micrometer. (b) Quantification of the number of NPCs around the CP in replicative Nup138::SmHA, Nup221::3xHA/GFP, and Nup313::SmHA parasites. Average is shown by the large data point. Nup138 *n* = 63 CP, Nup221 *n* = 95 CP, and Nup313 *n* = 81 CP. (c) Quantification of the number of NPCs around the CP in forming Nup138::SmHA, Nup221::3xHA/GFP, and Nup313::SmHA merozoites. Average is shown by the large data point. Nup138 *n* = 28 CP, Nup221 *n* = 17 CP, and Nup313 *n* = 26 CP.

During and after the first mitosis, we observed a random distribution of NPCs around individual nuclei, as visualized by Nup138 and Nup313 (Fig. 2; Fig. S2), where there does not appear to be a specific arrangement of NPCs. Interestingly, upon the first round of mitosis we detected 4–10 foci of Nup138, Nup313, and Nup221, the homolog to yeast Nup100/116/145N, localizing around the centriolar plaque (CP), which is the nuclear microtubule organization center (MTOC) responsible for intranuclear microtubule nucleation (Fig. 3a) (25, 31, 32). Quantification of NPCs around the centriolar plaque reveals an average of between five and six Nups, found in a rosette shape around the CP (Fig. 3a and b). It is important to note that after parasite cytokinesis, the centriolar plaque disassembles (25) (Fig. 3a, merozoites), but NPCs retain a rosette formation near the site of the former centriolar plaque (Fig. 3c). Interestingly, even after the CP disassembles, approximately 30% of NPCs in the daughter merozoites remain located near the inner region where the CP was previously positioned.

### The RITE system reveals NPC assembly and maintenance

**Fig 4:**
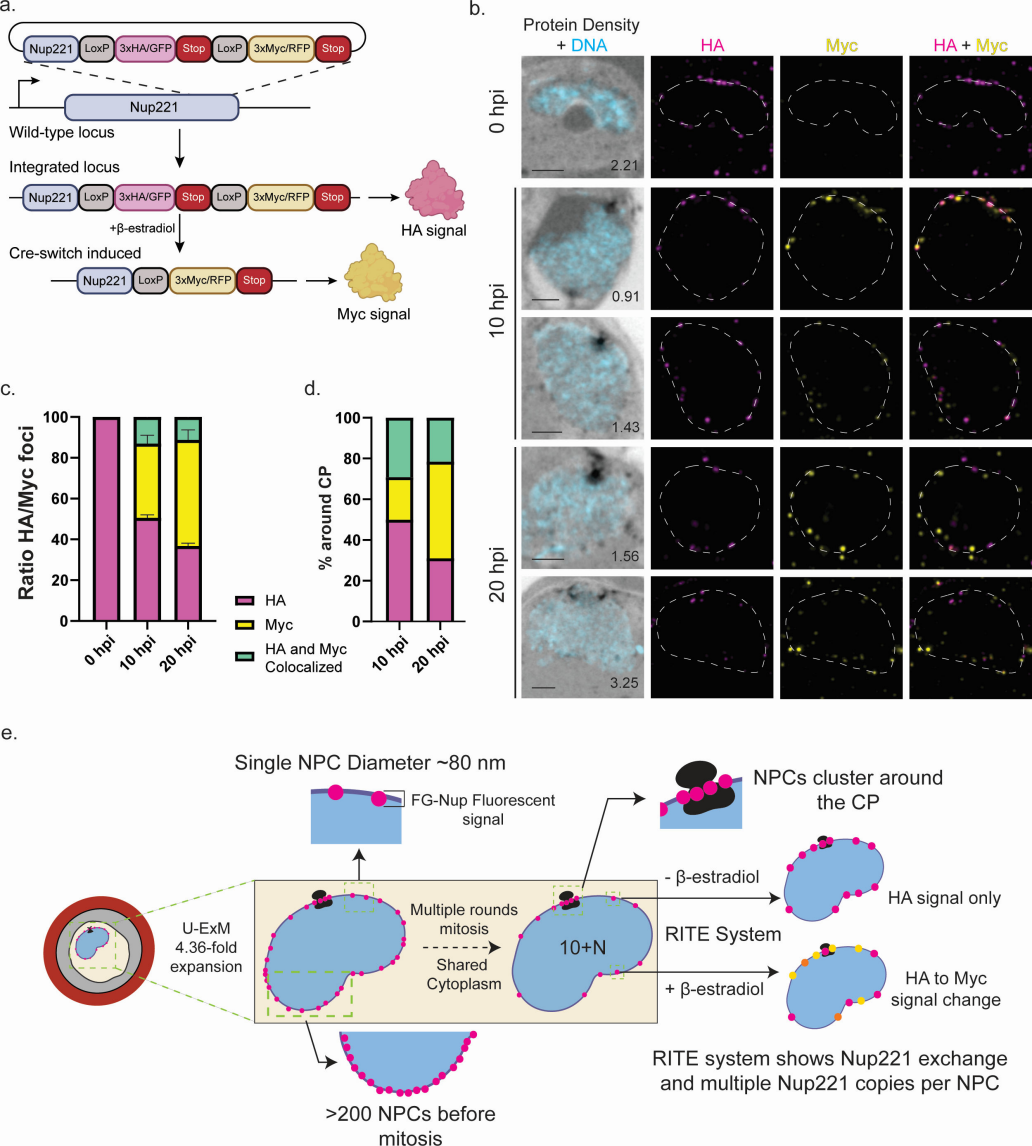
The RITE system shows Nup221 protein exchange in established NPCs. (a) Schematic showing endogenous tagging strategy to introduce the RITE system at the 3′ end of the *Nup221* gene. Translocation of Cre-EBD to the nucleus following treatment with β-estradiol results in excision between loxP sites, removing the original 3xHA/GFP tag while bringing the 3xMyc/RFP tag into frame. (b) Representative images of *P. berghei* parasites at 0, 10, and 20 hours post-induction with β-estradiol. HA signal is shown in magenta, and Myc signal is shown in yellow. Scale bars 2 µm; the number on images indicate image depth in micrometer. (c) Ratio of Nup221::HA/Nup221::Myc foci in 0, 10, and 20 hours post-induction with β-estradiol. Green bars indicate the number of foci that had both HA and Myc signals. *n* = 132 nuclei from 39 parasites across both conditions. (d) Amount of HA or Myc signal found around the CP in 10 and 20 hours post-induction parasites determined by counting foci around the CP. Green bars indicate foci containing both HA and Myc signals. (e) Schematic representation of the study presented in this manuscript. *P. berghei* parasites start as single nucleus parasites, with NPCs measured to be ~80 nm and a population of NPCs counting greater than 200 before mitosis. After multiple rounds of asynchronous mitosis, the NPCs produced are shared between the new nuclei, where they show a clustering around the CP. Finally, using the RITE system, we were able to show Nup221 protein exchange in NPCs as well as multiple copies of Nup221 protein in a single NPC, similar to what has been shown in other organisms.

NPCs in yeast undergo degradation and replacement of Nups and full NPCs (33–35), and Nup replacement occurs in non-dividing human cells (27). Therefore, to assess the turnover of Nups in *Plasmodium*, we adapted the RITE system for the first time in these parasites. The inducible RITE system uses a constitutively expressed Cre recombinase fused to the human estrogen binding domain (EBD), which retains the protein in the cytoplasm by association with HSP70. Upon the addition of β-estradiol, Cre-EBD translocates to the nucleus, where it triggers a permanent genetic switch through a recombination event between two *LoxP* sites: one positioned immediately upstream of the initial tag, removing this tag and bringing a distinct tag into frame (28) (Fig. S4a). Here, we tagged *P. berghei* Nup221 with a RITE system cassette, which includes an initial 3xHA/GFP tag, a second 3xMyc/RFP tag, and the two *LoxP* sites for tag switching (Fig. S2; Fig. 4a). We found that adding as little as 2 nM β-estradiol resulted in a partial switch from HA to Myc tag. In comparison, 200 nM β-estradiol induces sufficient relocation into the nucleus for a complete switch from HA to Myc tag in as little as 2 hours, confirmed by PCR analysis (Fig. S4b and c).

Initial immunofluorescence imaging over the course of a 16-hour *in vitro* culture period for both control and 200 nM β-estradiol treated parasites showed visible tag switching by 8 hours post-induction with β-estradiol (Fig. S5a). By the end of the 16-hour period, we observed individual parasites (newly formed, differentiated merozoites) that expressed a mixture of Nup221 tagged with GFP or RFP, both in a typical polar localization. By PCR genotyping, we can detect tag exchange within 2 hours of estrogen addition. Notably, the continued detection of Nup221-HA through 16 hours post-induction indicates a stable population of Nup221 that persists for at least 8 hours during the parasite life cycle (Fig. S4). To confirm complete replacement of Nup221 and rule out spurious excision, samples from control and induced conditions were propagated for an additional 96 hours (four parasite development cycles) *in vivo*. Myc-tagged Nup221 was never detected in control samples, confirming the strict requirement of estrogen to induce the tag exchange over that time period. After 96 hours, HA-tagged Nup221 was no longer detectable in β-estradiol-treated parasites. These results demonstrate that the RITE system is an effective tool for monitoring Nup221 turnover in *P. berghei*. Pairing this technique with U-ExM will enhance our ability to determine NPC maintenance in the parasites.

### *Plasmodium berghei* replaces Nups in post-mitotic NPCs

To investigate NPC assembly and maintenance during and after mitosis, we treated newly invaded Nup221 parasites (1N) with 200 nM β-estradiol and cultured for 20 hours. We then collected processed samples for U-ExM at 0, 10, and 20 hours post-induction with β-estradiol to assess Nup221 presence in NPCs before mitosis occurs (10 hours post-induction), after mitosis, and during cytokinesis (20 hours post-induction). We hypothesized that if Nup221 is being replaced in the post-mitotic NPCs, we would observe a shift in the signal from the original HA signal (pink) to the induced Myc signal (yellow), with a higher proportion of induced signal in the 20 hours post-induction samples.

At 0 hours post-induction, early-stage parasites exclusively expressed the original HA signal (Fig. 4b). By 10 hours post-induction, as trophozoites matured and initiated the first rounds of mitosis, resulting in the formation of two to three nuclei, these parasites exhibited a mix of the HA and Myc signals: 50% of foci contained only the original HA signal, 35% of foci contained only the new Myc signal, and 15% of foci contained both the HA and Myc signals (Fig. 4b and c). The co-localization of HA and Myc signals provides strong evidence for multiple copies of Nup221 in individual NPCs. Additionally, these chimeric NPCs with both “old” and “new” Nup221 suggest that NPCs can be formed not only *de novo* but may also result from NPC division or replacement of individual Nup221 within an existing NPC. By 20 hours post-induction with β-estradiol, the parasites ceased producing new NPCs, and the existing NPCs were divided among the daughter progeny. At this stage, we found that 35% of NPCs contained only HA signal, 50% of NPCs contained only the Myc signal, and 15% of NPCs contained both HA and Myc signals (Fig. 4b and c). This result suggests that novel Nup221-Myc is incorporated into existing NPCs after the parasites have stopped producing new ones.

We then wondered whether the Nups in the NPCs surrounding the CP were maintained at the same rate as those around the nucleus. To test this, we specifically monitored the turnover of Nup221 within the NPCs surrounding the CP. At 0 hours post-induction, when no CPs are visible, Nup221 is evenly distributed around the parasite nucleus. By 10 hours post-induction, the CP is present, and 50% of the NPCs surrounding it contained only the original signal, similar to what was observed around the rest of the nucleus. Approximately 20% of the NPCs contained only the induced signal, which is 15% lower than those around the nucleus, and 30% of the NPCs contained both the original and induced signal, 15% higher than those around the nucleus (Fig. 4d). This suggests that the turnover of the original Nup221 signal occurs at a slower pace in the NPCs surrounding the CP compared to those scattered around the nucleus. This trend persists at 20 hours post-induction, where 30% of NPCs showed only the original signal, consistent with the observations around the rest of the nucleus. However, 45% of the NPCs contained only the induced signal (a decrease from 50%) and 25% of the NPCs contained both the original and induced signals (an increase from 15%) (Fig. 4d). Altogether, our data indicate that proteins in the NPCs surrounding the CP exhibit greater stability than those in NPCs scattered around the nucleus.

## DISCUSSION

In this study, we applied U-ExM and the RITE system to investigate the distribution and homeostasis of NPCs in the asexual blood stages of the rodent malaria model, *P. berghei*. Previous work on Nups in *Plasmodium* has been challenging due to the divergent nature of the Nups and the relatively small size of the parasite nucleus, approximately 1 µm. Our results demonstrate U-ExM’s capability to resolve individual NPCs, allowing us to explore their individual characteristics. Additionally, we successfully adapted the RITE system in *Plasmodium* for the first time*,* allowing us to dissect the mechanisms of NPC maintenance during the parasite cell cycle. While we used the RITE system here to monitor protein turnover via tag exchange, it can also be adapted to other conditional mutagenesis strategies. This offers an alternative to the well-established dimerizable Cre system (36, 37), further expanding the genetic toolkit available in this important rodent malaria model.

This study quantified the number of Nups surrounding the nucleus during the asexual life cycle of *P. berghei*, revealing a dual localization of NPCs around individual nuclei. Notably, we observed a rosette structure around the CP during nuclear replication, which persisted in merozoites even after the CP was no longer visible (Fig. 3a and c). Our findings are consistent with those of Ferreira et al. (38), who used CryoEM to examine microtubules in *P. falciparum* and discovered similarly organized NPC structure around the CP during mitosis. This circular arrangement, previously unreported and unique to *Plasmodium*, may indicate a specific role of these NPCs in CP formation, duplication, maintenance, and function during mitosis. For example, in *S. cerevisiae*, the NPC protein Ndc1, a subunit of the transmembrane ring, plays a critical role in inserting the nuclear MTOC into the nuclear envelope (39, 40). However, this protein appears to be absent in *Plasmodium* and related apicomplexan parasites (VeuPathDB BlastP search with yeast Ndc1, Uniprot P32500).

By quantifying individual Nup foci around the nuclei, we determined the number of NPCs per nucleus throughout the life cycle. Our results indicate a rapid increase in NPCs just before the first round of mitosis, consistent with previous observations *in P. falciparum* (21). The higher NPC count observed in our study (250 vs 60) might be attributed to greater sample size achievable with U-ExM compared to FIB-SEM, as well as the improved NPC detection through immunostaining against endogenously tagged Nups (our data were derived from over 150 nuclei from over 60 cells across two biological samples).

Previous research across cell types has shown that the composition of NPCs at specific stages in the cell cycle can influence cell fate (41, 42). In *Plasmodium* parasites, it has been shown that SEC13, a component of the nuclear pore complexes and the COPII coat, displays different localization patterns compared to FG Nups (15). Our study explored NPC heterogeneity by examining three FG-Nups: Nup138, Nup221, and Nup313. Notably, Nup313 exhibited distinct localization in the early ring stage of the parasite, suggesting a specialized role during this stage of development. Further investigations into the dynamic localization of different types of Nups would be valuable to determine whether Nup313’s temporal differential localization is unique or shared with other Nups in the NPC.

Our data indicate that most NPCs contain a single version of Nup221, whether pre-mitotic (Fig. 4c, Nup221-HA, pink) or post-mitotic (Fig. 4c, Nup221-Myc, yellow). However, some NPCs exhibited both pre- and post-mitotic proteins, suggesting a gradual turnover in response to protein degradation or damage (Fig. 4c). These observations align with findings using the RITE system by Toyama et al. (27) and others, which suggest an active mechanism for NPC maintenance in mammalian cells (27, 43). This could also relate to the octagonal rotational symmetry observed in NPCs in other organisms (44, 45). Interestingly, we observed a slight increase in protein stability around the CPs, as evidenced by higher colocalization between Nup221-HA and Nup221-Myc proteins compared to the rest of the nucleus (Fig. 4d). This suggests greater longevity of this FG Nup in NPCs rosette compared to those scattered across the nucleus.

With the identification of numerous Nups in the *Plasmodium* genome (15, 17), and improved tools for visualizing NPCs, we are now positioned to investigate the individual functions of these Nups. Although the exact roles of specific Nups in the *Plasmodium* parasites remain unknown, studies in other organisms have highlighted the involvement of FG Nups in various cellular processes, including protein synthesis (46), NPC localization and composition (47, 48), as well as RNA transport (6, 49). While no studies have yet explored the specific function of FG Nups in *Plasmodium* parasites, genome-wide screens and individual knock-out attempts have shown that all Nups are essential (50–52). Similarly, partial knockdown of SEC13 in human malaria parasites slows their growth in the blood stage (16), and a C-terminal proline-enriched sequence in SEC13 is required for parasite growth in mouse models (15). Therefore, disrupting Nups is likely to result in growth delays and impaired cell viability in malaria parasites. Our work, along with previous studies, lays the foundation for future research into these divergent Nups as techniques for efficient knockdown continue to advance in malaria parasites.

In mammalian cells, one of the hallmarks of nuclear envelope breakdown during mitosis is the disassembly of NPCs following mitotic kinase signaling (53, 54). This disassembly is a critical step, where multiple Nups are phosphorylated and sequestered for use during nuclear envelope reassembly (55). In contrast, *Plasmodium* parasites, like yeast, undergo closed mitosis, where the nuclear envelope remains intact during nuclear division (13). Recent work in fission yeast has used confocal microscopy (11) and expansion microscopy (30) to examine the distribution and dynamics of NPCs in a closed mitosis model. These studies identified NPCs at the bridge between dividing nuclei before nuclear envelope fission and hypothesized that NPC disassembly in the bridge contributes to nuclear envelope disassembly, enabling nuclear division. While we observed instances of dividing nuclei with clusters of NPCs along this bridge, further investigation is needed to explore this mechanism in *Plasmodium* parasites. Identifying and characterizing a cytoplasmic-facing Nup would allow for a deeper exploration of this hypothesis and the mechanisms determining the location of NPC disassembly and its link to nuclear fission.

However, this study also highlighted certain limitations in NPC research in *Plasmodium*, as well as the limitations of U-ExM. While U-ExM allowed us to resolve individual NPCs around the nucleus, this technique does not distinguish the localization of specific Nups within individual NPCs. Recently, advances in expansion microscopy have emerged to allow for 16-fold expansion of samples compared to the 4.5-fold expansion seen by U-ExM (56). This iterative ultrastructure expansion microscopy has already been used to reveal the eightfold symmetry of human NPCs by visualizing Nup96-GFP, demonstrating differences between inner and outer ring Nups in human NPCs. This suggests that similar distinctions could be made with *Plasmodium* NPCs using this method. It is also important to note that this study focused on three of the five confirmed Nups in *Plasmodium berghei*, all of which are classified as FG-Nups. Further work is needed to identify structural Nups in the *Plasmodium* genome to distinguish between the nuclear and cytosolic sides of the NPC. Finally, we also noticed a non-specific signal found most commonly in the many food vacuoles present in *P. berghei*. However, these signals were consistently weaker than the true NPC signals, allowing us to distinguish between Nup signals on the nuclear membrane and the background signal. This excess signal has been reported in previous studies, and efforts are ongoing to reduce or eliminate this background noise.

In conclusion, our study provides novel insights into the distribution, homeostasis, and homogeneity of NPC complexes in *P. berghei* throughout the asexual life cycle. Understanding the dynamics of these essential components is crucial, as NPCs play a vital role in parasite survival. The methodologies employed here pave the way for further in-depth investigations into the functions of these divergent complexes, facilitating a comprehensive understanding of their role in *Plasmodium* biology. This study sets the stage for future investigations into the diverse Nups in the *Plasmodium* genome and their individual functions. Disruption of Nups, whether structural or FG Nups, is likely to impact cell growth and viability in malaria parasites, as observed in related studies. As techniques for improving knockdown efficiency continue to evolve, exploring the specific functions of Nups in *Plasmodium* is an exciting and imminent direction for research.

## MATERIALS AND METHODS

### Parasite maintenance

*P. berghei* ANKA clone 2.34 and derivatives were maintained in Swiss Webster mice (Charles River).

### Genetic modification of *Plasmodium berghei*

Plasmid cloning was carried out using NEBuilder HIFI DNA assembly (NEB). Primer sequences are given in Table 1. To generate a smHA (57) fusion at the endogenous C terminus of *P. berghei* Nup138, the smHA coding sequence was amplified from plasmid pCAG_smFP_HA (Addgene #59759) using primers P4121/4122 and inserted between BamHI/BsiWI immediately downstream of the *Pbnup138* homology region in the plasmid pLIS0654 (17) resulting in the plasmid pSA026. To generate a smHA fusion at the endogenous C terminus of *P. berghei* Nup313, a homology region corresponding to the 3′ coding sequence of *Pbnup313* up to but not including the stop codon was amplified with primers P4140/4126 from *P. berghei* genomic DNA and inserted between AvrII/BamHI in pSA026, replacing the *Pbnup138* homology region and resulting in the plasmid pSA028.

**TABLE 1:** Sequences of primers used in this study

Name	Sequence
P4121	GTTATTCTCACGAGCTTAGAAATATGAAGGGATCCATGTACCCTTATGATGTGCCCGATTATGC
P4122	GATAGCCAGCGTAGTCCGGGACGTCGTACGGGTAGCCACCCTTGTAGAGTTCGTC
P4140	GACGATAAAAATGAAAATACAAATCCTAGGGAAATTGAAATCGCTTGCACATATTTGAATC
P4126	CATCATAAGGGTACATGGATCCGTTTATGACATTTTGGCTAGTG
P5	GGAAAAAGCTTAACATTTAACTCTTCATTTTTTAAAACGAGCAC
P6	CCGGGACGTCGTACGGG
P7	CTTAGCCCATGCGAATGCATACTATTAGAAC
P8	GTCCATTAACGTCGCCATCCAACTCC
P3922	TTTAAATGATACAAAACACAGACG
P3915	ATACTAGTAGCGTAATCTGGAACG
P3888	TCAATGATTCATAAATAGTTGGACTTG
P3659	ATCAACAAATTATAAATGACAGAAC
P3004	AAAAGATCTATGGTTGGTTCGCTAAACTG
P3005	AAACAATTGTTAATCATTCTTCTCATATAC
P3491	GATAAATTCTCGAGCCCGGGTATCGATTATTTAGAGAATC
P3492	TCTTTACCTCCGCCGGATCCAATATTTTTAATAACATTTC
P4046	TTCCATGACCACCTTCATACGC
P3921	CGCATGAACTCCTTGATGACG

To generate parasites expressing Cre-EBD with the RITE cassette fused to the endogenous C terminus of *P. berghei* Nup221, the Cre-EBD coding sequence was amplified from the plasmid pTWO40 (58) (Addgene #64769) and the RITE cassette was amplified from the plasmid pKV016, which includes a 3xHA-GFP fusion in the pre-excised state and a 3xMYC-mRFP fusion in the post-excised state (59) (Addgene #64767). These sequences were assembled with a *Pbnup221* homology region (17), placing Cre-EBD under the control of the *P. berghei* EF1 alpha promoter and the hDHFR selectable marker under the control of the *P. berghei* EF1 delta promoter and positioning the hDHFR cassette between the two loxP-containing RITE tags to facilitate marker removal upon excision. The mRFP sequence in the post-excision RITE fusion was subsequently replaced with mRuby3 to yield the final plasmid pL857.

Plasmids pSA026, pSA028, and pL857 (Table 2) were linearized within the *Pbnup138*, *Pbnup313*, or *Pbnup221* homology region using HindIII, PacI, or ScaI, respectively, and transfected into *P. berghei* as described (60). Transfected parasites were injected into naive mice and selected with 0.07 mg/mL pyrimethamine provided *ad libitum* in drinking water beginning 24 hours post-transfection. Parasites were genotyped to confirm integration at *Pbnup138*, *Pbnup313*, or *Pbnup221* using PCR strategies described in Fig. S2 and S4.

**TABLE 2:** Plasmids used in this study

Genotype	Plasmid name
nup138::smHA	pSA026
nup313::smHA	pSA028
nup221::RITE	pL857

### Live imaging and immunofluorescence assays

For live imaging of parasites in supplemental figures, at regular intervals throughout the experiment, 100 µL parasite culture was spun down and resuspended in PBS containing 1 µg/mL Hoechst 33342 to stain nuclei. Following a brief incubation at 37℃, parasites were imaged with a 63× objective on an Axio Observer 7 equipped with an Axiocam 702 mono camera and Zen 2.6 Pro software (Zeiss) using the same exposure times for all images across sample groups and experimental replicates. For immunofluorescence assays on unexpanded cells, thin blood smears were air dried and fixed in ice-cold methanol/acetone (50:50) (for the detection of smHA-tagged Nup138, Nup313, or Cre). Fixed slides were blocked with Rockland blocking buffer (Rockland) and probed with the primary and secondary antibodies indicated below. Slides were mounted in Fluoromount-G with DAPI (Invitrogen).

### Ultrastructure expansion microscopy

Ultrastructure expansion microscopy was performed as previously described on fixed samples. Briefly, parasites were fixed in 4% PFA/PBS supplemented with 0.0075% glutaraldehyde by the lab of Dr. Josh Beck at Iowa State University and were sent to the lab of Dr. Sabrina Absalon at Indiana University School of Medicine. Twelve-millimeter round coverslips (Fisher Cat. No. NC1129240) were treated with poly-D-lysine for 1 hour at 37°C, washed three times with MilliQ water, and placed in the wells of a 24-well dish. Fixed samples were resuspended at roughly 1.0% hematocrit in 1× PBS, and 500 µL of parasite sample was added to the wells containing the coated coverslips for 30 minutes at 37°C. Following parasites settling, coverslips were washed three times in 1× PBS before treating with 500 µL 1.4%, vol/vol formaldehyde/2%, vol/vol acylamide in PBS. Samples were incubated overnight at 37°C. Gelation, denaturation, staining, and expansion of the gels were performed as previously described (24). Stained gels were imaged using a Zeiss LSM900 AxioObserver with an Airyscan 2 detector. Images were taken using a 63× Plan-Apochromat objective lens with a numerical aperture of 1.4.

### RITE experiment setup

Asynchronous Nup221::RITE *P. berghei* parasites were collected and allowed to develop overnight *in vitro* into terminal schizonts. Schizonts were magnetically purified on a MACS LD column (Miltenyi Biotec) and IV injected into naive mice to establish a synchronous infection. Infections were allowed to develop for three intraerythrocytic developmental cycles (IDCs) to increase parasitemia, then collected at 72 hpi when parasites were predominantly ring stage and passed through a MACS LD column to remove any trophozoites or schizonts by collecting the flow through.

These synchronized ring-stage parasites were used to establish *in vitro* cultures in complete RPMI containing 200 nM β-estradiol or DMSO (vehicle control). Parasites were collected every 2 hours for live imaging and genomic DNA isolation. After 24 hours, parasites were washed to remove β-estradiol or DMSO and IV injected into naive mice to monitor Nup turnover in subsequent IDCs. Mice were not provided with pyrimethamine to avoid killing parasites that had undergone excision, which also removes the hDHFR selection marker.

### Antibodies and stains

The following antibodies were used for widefield IFA analysis: rabbit polyclonal anti-HA (1:500, Thermofisher SG77) and rabbit anti-Cre polyclonal (1:500, Abcam ab190177). The following antibodies were used for the preparation of stained gels for expansion microscopy: Rat anti-HA (3F10, 1:25, Roche 12158167001), Mouse IgG1 anti-alpha Tubulin Clone B-5-1-2 (1:500, ThermoFisher 32-2500), and Rabbit anti-Myc polyclonal (1:500, ThermoFisher 710007). Goat anti-rabbit Alexa Fluor 594 (1:500, ThermoFisher A11012), Goat anti-rat Alexa Fluor 488 (1:500, ThermoFisher A1106), Goat anti-mouse IgG(H + L) Alexa Fluor 555 (1:500, ThermoFisher A21422), and Goat anti-rabbit Alexa Fluor 555 (1:500, ThermoFisher A32732) were used as secondary antibodies. NHS Ester Alexa Fluor 405 in DMSO (8 µg/mL, ThermoFisher A30000) was used to stain proteins, and SYTOX Deep Red (1 µM, ThermoFisher S11380) was used as a DNA stain.

### Image analysis and NPC quantification

All image analysis was done using 3D Airyscan processing at moderate filter strength on Zen Blue version 3.1 (Zeiss, Oberkochen, Germany). Images shown in this manuscript are maximum intensity projections of between 5 and 60 z-slices of the entire image, with the number indicated as the total *Z* depth of the image (*Z* depth = 0.13 µm × number of slices taken for projection). NPCs were quantified by setting manual thresholds and counting the foci that appear at the nuclear periphery manually. Signals found distant from the nuclear periphery but visible after thresholding were excluded as likely unspecific staining. NPC size was determined as previously described in reference (30). Briefly, a line was drawn over the Nup foci, and the distance between the half maximum intensity of the signal was determined using Zen Blue version 3.1.

### Statistical analysis

Graphical data and statistical analysis were performed on GraphPad Prism version 10.3.0.

## SUPPLEMENTAL MATERIAL

Legends msphere.00750-24-s0001.docx Legends for the supplemental figures and tables.

Table S1 msphere.00750-24-s0002.xlsx Nup counting.

Table S2 msphere.00750-24-s0003.xlsx NPC diameter.

Fig. S1 msphere.00750-24-s0004.tif Nuclear envelope visualization in *P. berghei*.

Fig. S2 msphere.00750-24-s0005.tif Genotyping of Nup138, Nup313, and Nup221 endogenously tagged parasite lines.

Fig. S3 msphere.00750-24-s0006.tif Nup313 through the *P. berghei* blood stage life cycle

Fig. S4 msphere.00750-24-s0007.tif Cre-EBD enables inducible genomic excision in *P. berghei*.

Fig. S5 msphere.00750-24-s0008.tif Time course of the RITE system in *P. berghei* of Nup221::RITE.

ASM does not own the copyrights to Supplemental Material that may be linked to, or accessed through, an article. The authors have granted ASM a non-exclusive, world-wide license to publish the Supplemental Material files. Please contact the corresponding author directly for reuse.
