# Supplementary material for: Nuclear pore complexes undergo Nup221 exchange during blood-stage asexual replication of Plasmodium parasites
Source: mSphere. 2024 Nov 11;9(12):e00750-24. doi: 10.1128/msphere.00750-24 (PMC11656741; doi:10.1128/msphere.00750-24)
Supplement: Legends — for the supplemental figures and tables. [file msphere.00750-24-s0001.docx]

**Supplemental Figure Legends:**

**Fig. S1: Nuclear envelope visualization in *P. berghei*.** **a.** Size of all gels used for this study, averaging 52.4 mm, giving a 4.36x expansion factor for our study. **b.** Staining with the endoplasmic reticulum protein PfBip (top image, yellow), Bodipy TR ceramide and Bodipy FL ceramide to stain for membranes (middle images, white), and differences in NHS ester staining (grayscale) to visualize the nuclear envelope. Magenta indicates Nup138 or Nup313, scale bars 2 µm, number on images indicates image depth in µm.

**Fig. S2: Genotyping of Nup138, Nup313 and Nup221 endogenously tagged parasite lines.** **a.** Schematic of general strategy for integration into the endogenous Nup138, Nup313 and Nup221 loci to generate C-terminal tag fusions. The plasmid is linearized with the homology flank targeting each gene (yellow/red box). Following transfection, homologous recombination into the target locus (blue box) results in fusion of the tag to the 3’ end of the gene. Green and red lines show integration PCR products diagnostic for the successful integration. sm, selectable marker. **b & c.** 5’ integration PCRs for Nup138-smHA and Nup313-smHA parasites. **d.** Schematic of the plasmid used to generate the Nup221-RITE parasites. Cre-mediated recombination between loxP sequences removes the 3xHA-GFP tag and hDHFR selection marker and brings the mRuby3-3xMyc tag into frame. **e.** Integration PCRs for the Nup221-RITE parasites. Reactions 1 and 2 are diagnostic for successful 5’ and 3’ integration, respectively. Reactions 3 and 4 are controls amplifying an internal portion of the plasmid and an unrelated genomic locus, respectively.

**Fig. S3: Nup313 through the *P. berghei* blood stage life cycle.** U-ExM images showing the distribution of Nup313 through the life cycle of *P. berghei*. DNA shown in blue, microtubules shown in yellow, Nup313 shown in magenta, protein density shown in grayscale. All scale bars 2 μm, number on images indicates image depth in µm.

**Fig. S4: Cre-EBD enables inducible genomic excision in *P. berghei.*** **a.** Immunofluorescence of Cre-EBD expressing *P. berghei* parasites. The addition of β-estradiol to the parasites induces the Cre-EBD to translocate to the nucleus. Scale bar 2 µm. **b.** PCR showing genome shift upon addition of β-estradiol, inducing a tag switch. Primers used are indicated in the diagrams and expected length of PCR products are listed. **c.** PCR showing concentration dependence of the RITE system tested from 0.0002 µM to 200 µM β-estradiol. 2 nM β-estradiol is sufficient to induce a partial tag switch, while 200 nM β-estradiol was sufficient to induce a total tag switch after 2 hours of treatment. PCR primers used are listed in the diagram.

**Fig. S5: Time course of the RITE system in *P. berghei* of Nup221::RITE. a.** Live imaging of Nup221::RITE parasites showing control or β-estradiol treated parasites during 16 hours of treatment, samples taken every 2 hours. In addition, parasites were cultured an additional 80 hours, where β-estradiol treated parasites only showed RFP signal and control parasites still only showed GFP signal. Scale bars 2 µm. **b.** PCR gel showing the efficiency of Cre-EBD system over 16 hours. PCR primers and expected band sizes are indicated in the diagram.

**Table S1: Number of NPC foci around nuclei.** Tables showing the number of Nup138::smHA, Nup313::smHA, and Nup221::Rite system foci around nuclei in different parasites. Data also counts the number of foci seen around the centriolar plaque (CP) if there is one visible, and for the RITE system this also shows the number of GFP foci, followed by Myc foci, then colocalized GFP and Myc signal. Multiple sets of numbers represent multiple CPs in the same nucleus and the foci around them.

**Table S2: Diameter of NPCs measured.** Tables showing the diameter of Nup138::smHA, Nup221::Rite system, and Nup313::smHA foci around the nucleus. Diameter of the NPCs were measured by drawing a line over the foci and measuring the distance between the half maximal intensity of the signal (see Figure 2). Diameter of the pre-expansion foci estimated by dividing the diameter measured after expansion by the expansion factor measured at 4.36-fold for this study.
