# Supplementary figures and images for: Nuclear pore complexes undergo Nup221 exchange during blood-stage asexual replication of Plasmodium parasites
Source: mSphere. 2024 Nov 11;9(12):e00750-24. doi: 10.1128/msphere.00750-24 (PMC11656741; doi:10.1128/msphere.00750-24)

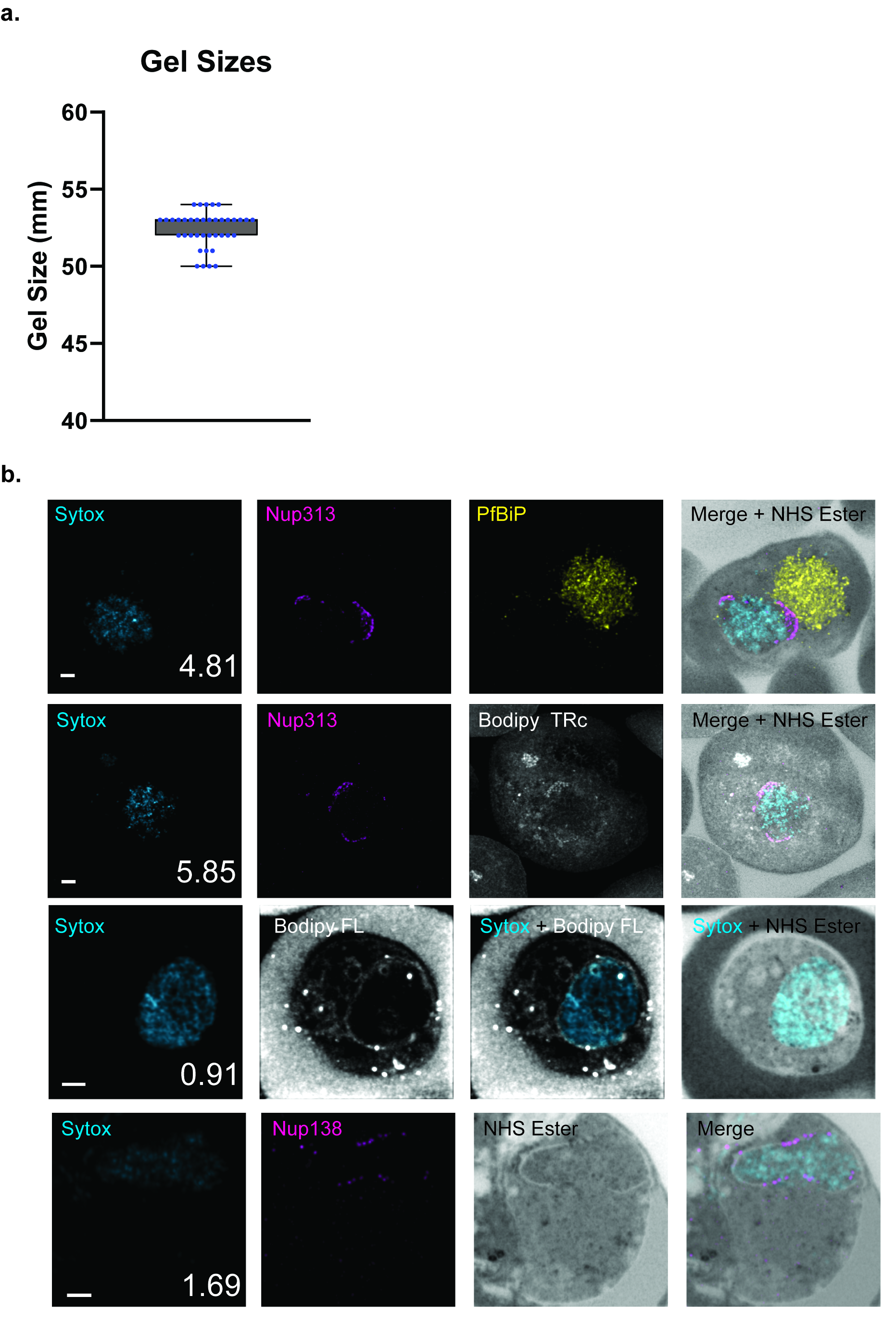

Supplement: Fig. S1 — Nuclear envelope visualization in P. berghei. [file msphere.00750-24-s0004.tif]

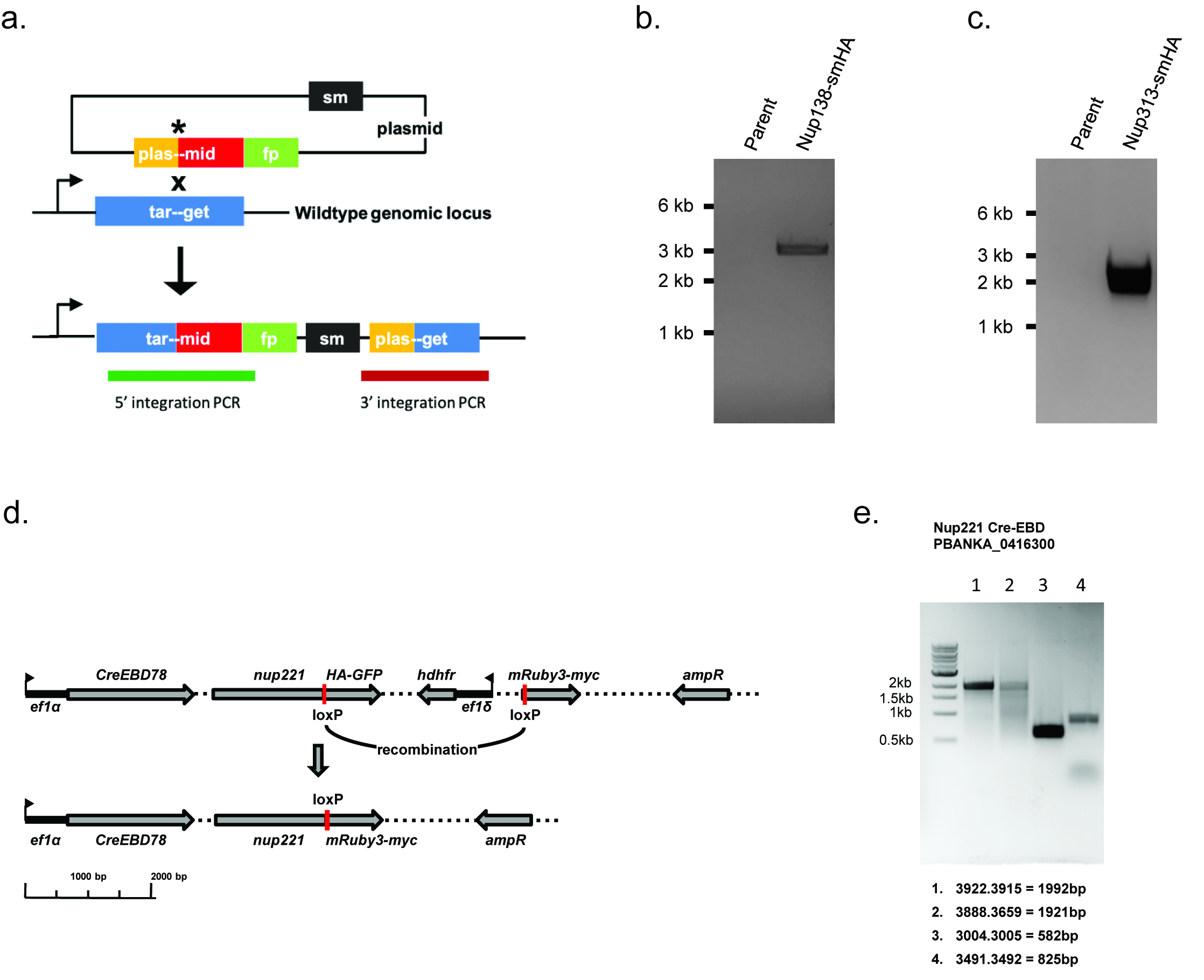

Supplement: Fig. S2 — Genotyping of Nup138, Nup313, and Nup221 endogenously tagged parasite lines. [file msphere.00750-24-s0005.tif]

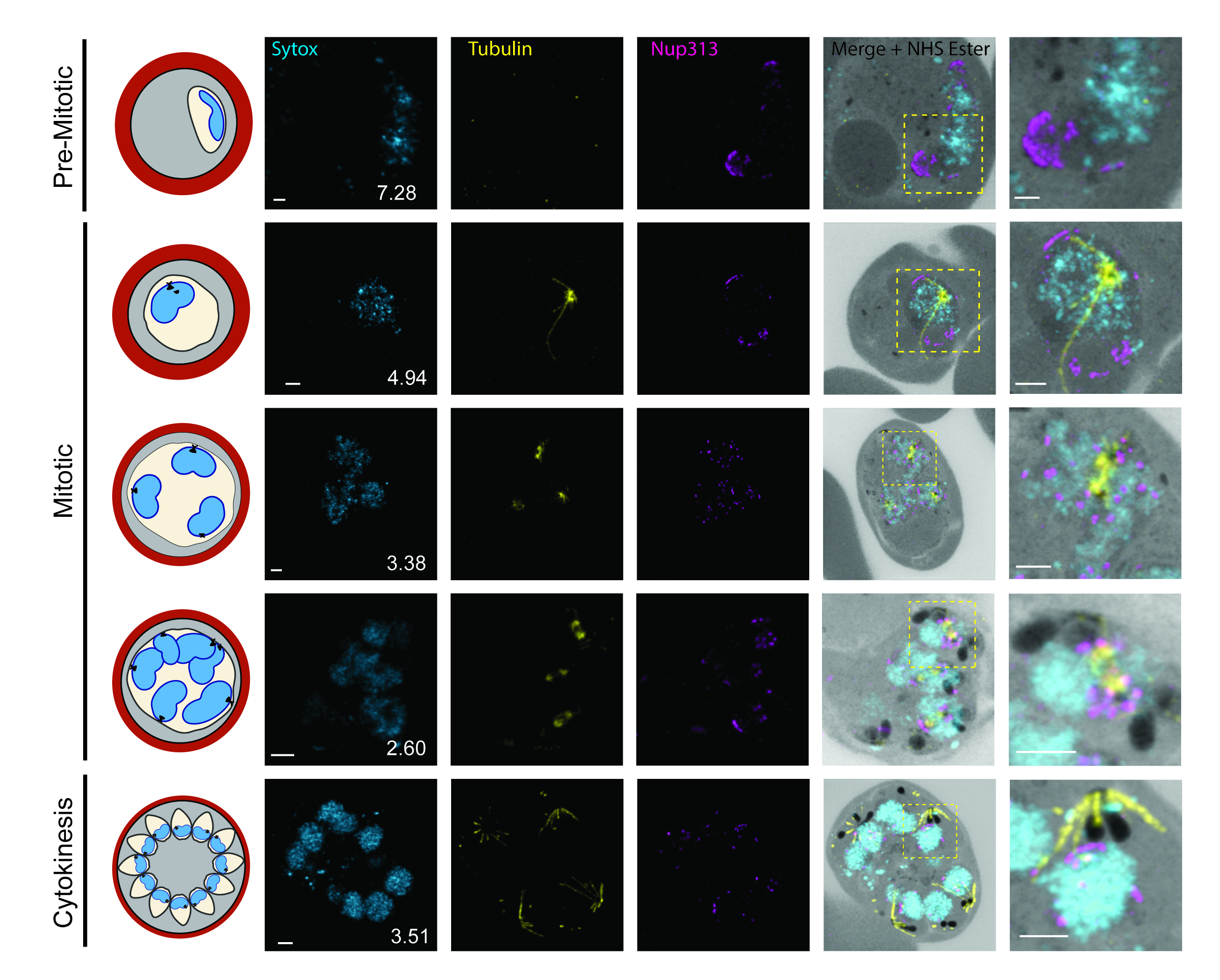

Supplement: Fig. S3 — Nup313 through the P. berghei blood stage life cycle [file msphere.00750-24-s0006.tif]

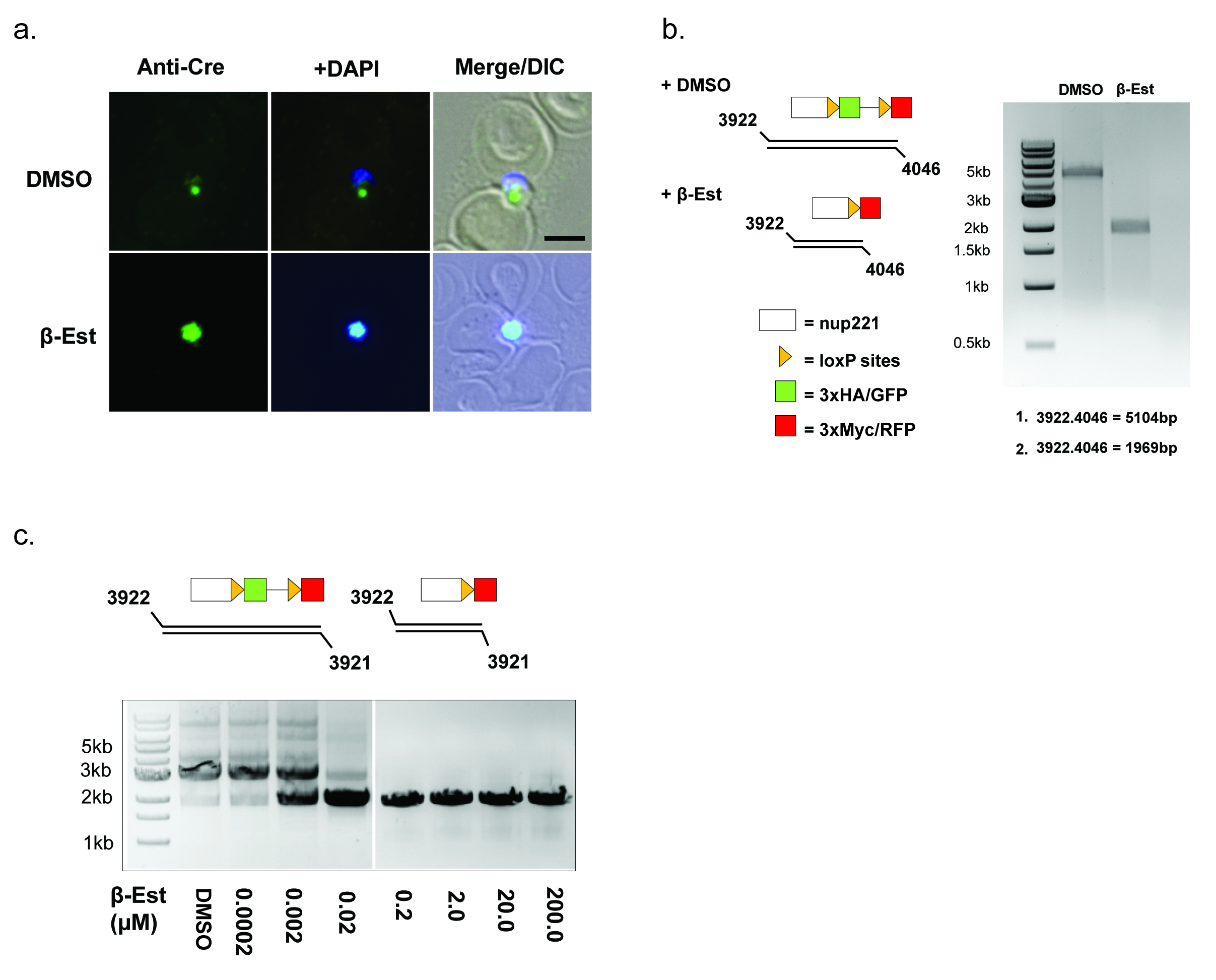

Supplement: Fig. S4 — Cre-EBD enables inducible genomic excision in P. berghei. [file msphere.00750-24-s0007.tif]

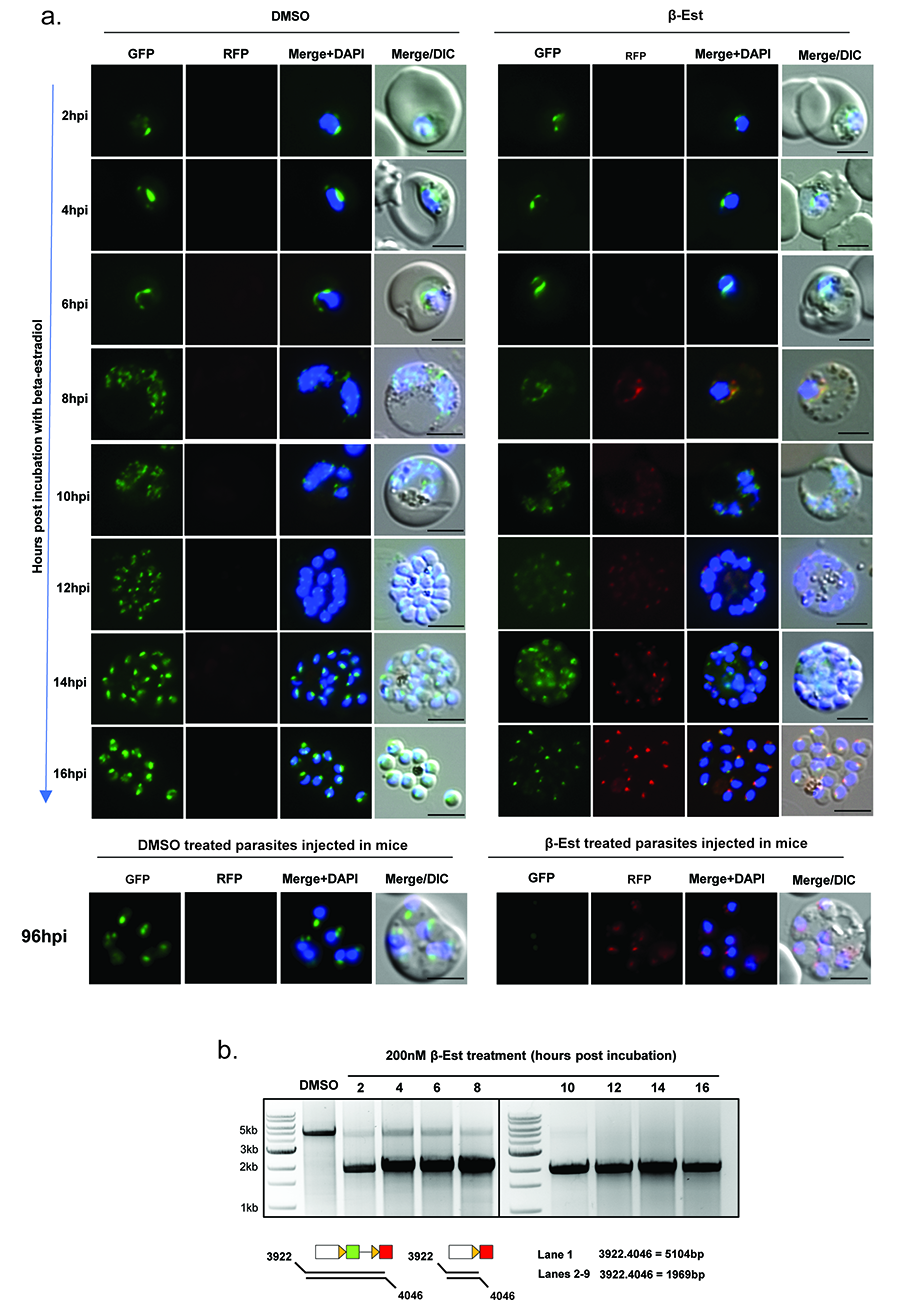

Supplement: Fig. S5 — Time course of the RITE system in P. berghei of Nup221::RITE. [file msphere.00750-24-s0008.tif]
